# Supplementary material for: pH-Sensitive Nanoparticles for Colonic Delivery Anti-miR-301a in Mouse Models of Inflammatory Bowel Diseases
Source: Nanomaterials (Basel). 2023 Oct 20;13(20):2797. doi: 10.3390/nano13202797 (PMC10610125; doi:10.3390/nano13202797)
Supplement: Supplementary file 1 [file nanomaterials-13-02797-s001.zip › nanomaterials-2638823-supplementary.pdf]

Supplementary information

pH-Sensitive Nanoparticles for Colonic Delivery Anti-miR-301a in Mice Models of Inflammatory Bowel Diseases

Junshan Wang [1,*], Min Yao [2], Jiafeng Zou [2], Wenxing Ding [2], Mingyue Sun [2], Ying Zhuge [3] and Feng Gao [2,4,5,6,*]

[1] Department of Gastroenterology, Chongming Branch of Shanghai Tenth People's Hospital, Tongji University School of Medicine, Shanghai 202157, China
[2] Shanghai Frontier Science Research Base of Optogenetic Techniques for Cell Metabolism, School of Pharmacy, East China University of Science and Technology, Shanghai 200237, China; ym1584007995@163.com (M.Y.); zoujiafeng2019@126.com (J.Z.); dwx150120413@gmail.com (W.D.); 17854280576@163.com (M.S.)
[3] Department of Cardiology, Shanghai General Hospital, Shanghai Jiao Tong University School of Medicine, Shanghai 200025, China; yiliaobangzu@163.com
[4] Shanghai Key Laboratory of New Drug Design, School of Pharmacy, East China University of Science and Technology, Shanghai 200237, China
[5] Shanghai Key Laboratory of Functional Materials Chemistry, East China University of Science and Technology, Shanghai 200237, China
[6] Optogenetics and Synthetic Biology Interdisciplinary Research Center, State Key Laboratory of Bioreactor Engineering, East China University of Science and Technology, Shanghai 200237, China
* Correspondence: jswang22@126.com (J.W.); fgao@ecust.edu.cn (F.G.)

Supplementary Figures

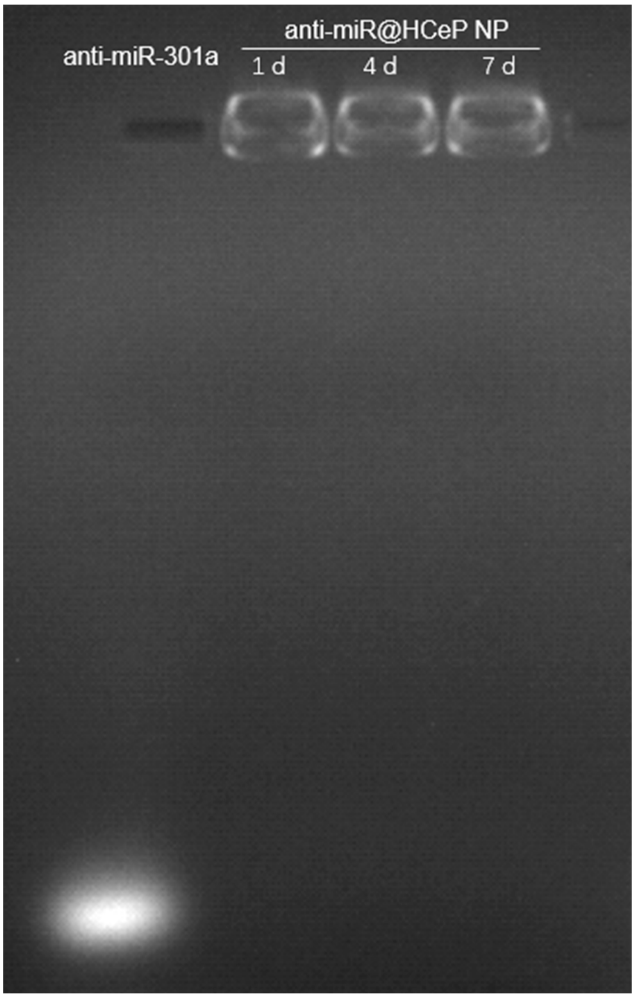

Figure S1. The stability of anti-miR@HCeP NP at 1, 4 and 7 d.

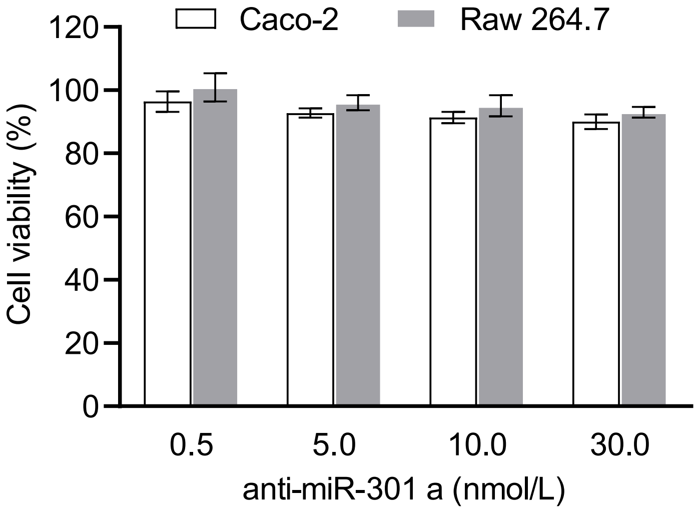

Figure S2. The cell viability of FAM-anti-miR@HCeP NP
